# Shaped by the Past: The Default Mode Network Supports Cognition that Is Independent of Immediate Perceptual Input

**DOI:** 10.1371/journal.pone.0132209

**Published:** 2015-06-30

**Authors:** Mahiko Konishi, Donald George McLaren, Haakon Engen, Jonathan Smallwood

**Affiliations:** 1 Department of Psychology & York Neuroimaging Centre, University of York, York, United Kingdom; 2 Department of Neurology, Massachusetts General Hospital and Harvard Medical School, Boston, United States of America; 3 Department of Social Neuroscience, Max Plank Institute for Human Cognitive Brain Sciences, Leipzig, Germany; Wake Forest School of Medicine, UNITED STATES

## Abstract

Although many different accounts of the functions of the default mode network (DMN) have been proposed, few can adequately account for the spectrum of different cognitive functions that utilize this network. The current study used functional magnetic resonance imaging (fMRI) to explore the hypothesis that the role of the DMN in higher order cognition is to allow cognition to be shaped by information from stored representations rather than information in the immediate environment. Using a novel task paradigm, we observed *increased* BOLD activity in regions of the medial prefrontal cortex and posterior cingulate cortex when individuals made decisions on the location of shapes from the prior trial and *decreased* BOLD activity when individuals made decisions on the location of shapes on the current trial. These data are inconsistent with views of the DMN as a task-negative system or one that is sensitive only to stimuli with strong personal or emotional ties. Instead the involvement of the DMN when people make decisions about where a shape was, rather than where it is now, supports the hypothesis that the core hubs of the DMN allow cognition to be guided by information other than the immediate perceptual input. We propose that a variety of different forms of higher order thought (such as imagining the future or considering the perspective of another person) engage the DMN because these more complex introspective forms of higher order thought all depend on the capacity for cognition to be shaped by representations that are not present in the external environment.

## Introduction

Understanding the function of the *default mode network (DMN)* has become an important question in cognitive neuroscience [1]. This core brain network, focused on hubs in both the medial prefrontal cortex (mPFC) and the posterior cingulate cortex (PCC), was initially identified via meta-analysis because of its tendency to deactivate during tasks that demand external focus [2]. Since then functional magnetic resonance imaging (fMRI) has shown that these regions show patterns of temporally correlated activity during the resting state [3] and comparative studies have identified an analogue of the DMN in many species including rats [4], chimpanzees [5] and macaques [6]. Developmental studies have shown this network matures relatively late in life in humans [7] and degeneration within key structures of the DMN occurs with age and is particularly prevalent in dementias such as Alzheimer’s Disease [8,9].

Such evidence indicates that the DMN plays an important functional role in cognition [1]. Task based studies suggest the DMN is involved in a wide variety of cognitive functions including semantic processing [10], thinking about oneself [11], imagining one’s future [12–15], encoding and retrieving episodic memories [16], retrieving autobiographical memories [17,18], considering the world from the perspective of another person [19,20] and thinking creatively about a problem [21–23] (for quantitative meta analyses see [24–27]).

The large number of functions utilizing the DMN has generated several hypotheses of this networks function. For example, the association between DMN and states of personally relevant processing has led to the suggestion that it supports information important for the autobiographical [28] or social aspects of the self [19]. The DMN has also been identified as important in task-irrelevant states such as mind wandering or daydreaming [29–35] and has been shown to lead to errors on tasks demanding external perception [36]. This latter point, coupled with the DMNs tendency to deactivate when performing such tasks [37], has led some authors to describe it as a *task negative network* [38–41] (although see [42]). Although the interpretations of the DMN as reflecting aspects of the self or as a task-negative system capture isolated aspects of the literature, they fail to provide an overarching account of the functions that this network performs. What is common, for example, to imaging the future or adopting another’s perspective and to deactivations during demanding perceptual tasks?

One possibility is that the DMN allows cognition to process representations that are not presently available to the senses, an account we will refer to as the *mnemonic facilitation hypothesis*. Two recent studies support this basic premise. Smallwood and colleagues [43] used a paradigm in which individuals made decisions using information from past trials or from information available on that particular trial. They found that under conditions when individuals were asked to recollect details of the parity of numerical stimuli, regions of the medial pre-frontal cortex and the posterior cingulate cortex exhibited greater activity when this judgement was made rapidly rather than when it was made slowly. The opposite pattern was observed when decisions were made when the stimulus was available to perception. More recently, Spreng and colleagues [44] explored the neural recruitment that occurs when participants perform a working memory task containing famous and non-famous faces. They found that the core aspects of the DMN show enhanced activity in a two back task when the target to be retrieved was a famous face, thus involving a combination of working memory and episodic memory. Together these two studies suggest that the role of the DMN in cognition is neither task-negative nor related to personally significant information but may reflect a system that supports a wide range of psychological states that depend on representations that are not available to the senses.

If a primary function of the DMN was to *allow* representations unrelated to perceptual input to guide thought and behaviour this could also explain why it is implicated in states of imagination that rely on memorial input for their content (such as thinking about the past or the future). Guiding thoughts and actions based on memory is also *unnecessary* for tasks that rely on a continuous focus on perception, explaining why the DMN often deactivates in tasks as the Eriksen Flanker task or Go–No Go tasks and why, under these conditions, its activity can be a cause of error [1,29,36,45]. The *mnemonic facilitation hypothesis* can also explain why aspects of the DMN are involved in semantic associative processes that depend on representations gained through experience [10] and why it exhibits activation when working memory targets are also encoded in long term memory (such as when they are a famous face) [44]. Importantly, it also explains the encoding-retrieval flip phenomenon where the DMN deactivates during encoding and activates during retrieval [16]. Relative to both the task-negative and autobiographical/social hypothesis, the *mnemonic facilitation hypothesis* leads to a simple prediction: *Regions of the DMN should be more engaged when a decision is made based on information represented in memory rather than immediate perceptual input*.

## Current study

**Fig 1:**
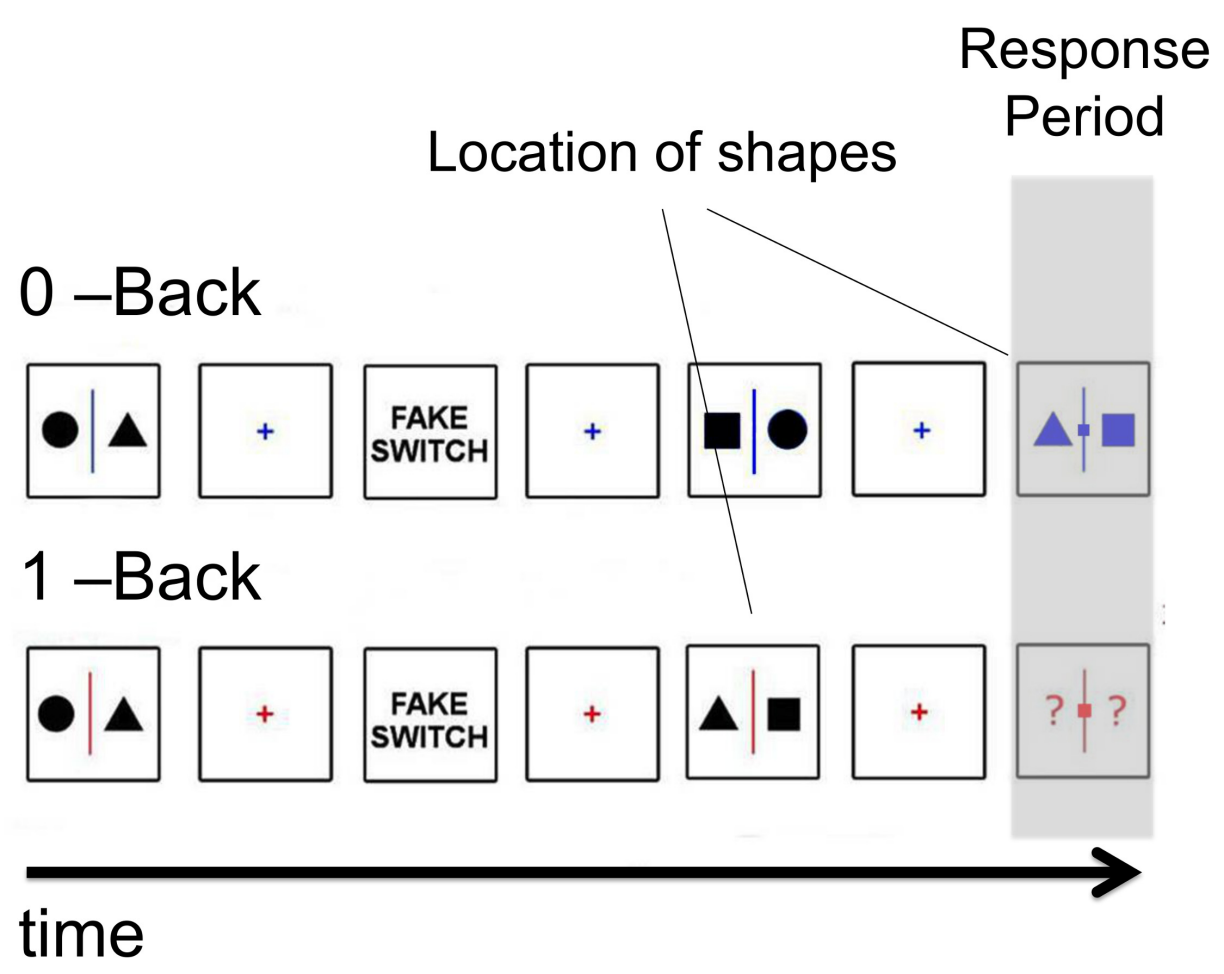
Experimental paradigm. Participants alternated between two tasks. One task involved observing non-coloured shapes presented at fixation waiting for the presentation of a coloured slide at which point they would indicate using a button press which side of the fixation cross a target shape was (0-back). In the other task participants had to encode the identity of shapes presented on screen and when prompted by a coloured slide to respond based on the position of a specific target shape on the prior trial (1-back). This paradigm requires participants to indicate the location of the same stimulus (for example the position of a square) which depends on whether the stimulus is immediately present or absent at the point at which the decision is made.

We developed a paradigm to test of our account of DMN function that builds on both our prior work and that of Spreng and colleagues. Participants alternate between task blocks in which they either make decisions about the location of shapes as they are presented on screen (0-back) or with respect to their location on the prior trial (1-back, see Fig 1). Engaging working memory reduces the occurrence of task unrelated thought [30,46–48]. Based on prior studies, therefore, we expected to replicate an increase in off task thoughts in the 0-back task and to find greater sustained activity in the DMN during this period because of its documented role in cognition that is generated by the individual [29–33].

Critical to the current investigation, however, is whether the DMN allows operations to be performed on previously acquired representations rather than that which is available to the senses. If it does, it should exhibit greater activity when decisions are made on the position of the shapes in the 1-back task relative to the 0-back task. Importantly because the judgment is regarding the spatial location of triangles, circles or squares, activation of the DMN under these conditions could not be attributed to the personal or emotional significance of the stimulus. Moreover because the 1-back task is more demanding than the 0-back task, greater DMN activity in this context could not be accounted for by the task-negative hypothesis. Finally, because we manipulate whether the same stimulus is available to perception or not, we can rule out differences in the role of long-term memory (such as familiarity with a famous face). To understand these questions we conducted a behavioural experiment to confirm that our modulation of working memory reduced off task thought and an fMRI experiment to explore the *mnemonic facilitation hypothesis* of DMN function.

## Methods

### Participants

#### Behavioral

Twenty-nine participants (9 males, age = 21.7±2 years) completed the behavioural study. Participants were recruited using the Psychology Electronic Experiment Booking System (PEEBS) of the University of York.

#### Task-based fMRI

Twenty participants (9 males, age = 23.8±3 years) completed the fMRI study. Participants were recruited using both PEEBS and the York Neuroimaging Center (YNiC) participants’ pool.

Both studies were approved by the Ethics Committee of the Psychology Department of the University of York. All investigation was conducted according to the principles expressed in the Declaration of Helsinki and for both studies participants provided written informed consent.

### Task paradigm

#### Behavioral

The task used in this experiment was programmed using PsychoPy [49]. The task featured a 0-back and a 1-back condition that continuously switched from one another throughout the experimental session (see Fig 1). Our paradigm is broadly similar to the paradigm used by Smallwood and colleagues [43] and was modified with the specific aim of maximising the differences between the 0-back and the 1-back conditions. In both conditions participants saw different pairs of shapes (Non-Targets, NT) appearing on the screen divided by a vertical line; the pairs could be: a circle and a square, a circle and a triangle, or a square and a triangle for a total of 6 possible pairs (two different left/right configurations for each). The pairs never had shapes of the same kind (e.g. a square and a square). In both tasks a block of NT was followed by target requiring participants to make a manual response. The target was a small stimulus presented in either blue or red and the colour was counterbalanced across participants. In the 0-back condition the target was flanked by one of two shapes and participants had to indicate by pressing the appropriate button which shape matched the target shape. In the 1-back condition, the target was flanked by two question marks and participants had to respond depending on which side the target shape was on the prior trial. For the behavioural study responses were made using the left and right arrow keys, for the neuroimaging study responses were made using a button box. Importantly, unlike the paradigm employed by Smallwood and colleagues [43] this design ensures that the participants cannot know what response to make when presented with the to be encoded stimulus.

Each block lasted between 40 to 120 seconds before switching to the other condition; the change of condition was signalled by a message (“SWITCH”) that remained on screen for 5 seconds. On each trial the number of NTs preceding the Targets varied between 2 and 6, the number of trials per block varied between 2 and 5 and the total number of blocks was 8 for each condition. The order of conditions was counterbalanced across participants and the whole task lasted ~35 minutes. The total number of targets was 15 to 20 per condition (0-back and 1-back). Also, in every block the word “STAY” was presented at the end of a trial indicating that participants were to remain in that condition. In order to sample the participants’ ongoing experiences we used a probe-caught, experience sampling method [50,51].The task was built so that there was a 50% chance of a thought probe being presented in place of a Target in a condition block and a maximum of one probe per condition block was allowed. The thought probe consisted in a screen prompting the participants to rate their focus level (“Where you focused on the task or were you thinking about something else?”) on a scale from 0 (completely off task) to 9 (completely on task).

Presentation rate of the stimuli was jittered in the following way. Fixation crosses ranged from 2–4 seconds in steps of 0.1s, Non-targets were varied from 1–3 seconds in steps of 0.1s. Targets always lasted a maximum of 4 seconds and a response from participants immediately ended the target presentation.

#### Task-based fMRI data acquisition

The paradigm used for the fMRI study was essentially the same with the following changes: there were no thought probes presented, fixation crosses were jittered between 2–3 seconds in steps of 0.1s, non-targets were jittered between 1–2 seconds in steps of 0.1s, targets stayed on the screen for 2.5 seconds regardless of a response being made by participants, switches and stay screens lasted 4 seconds, responses were made using an MRI compatible button box. The total number of blocks was 6 per condition in each run and the total number of targets was 8 to 12 per condition (0-back and 1-back) in each run, making it 12 blocks and 16 to 24 targets per condition in total.

Imaging was performed at the York Neuroimaging Centre (YNiC) using a GE 3.0 Tesla HDx Excite MRI scanner using an 8-channel head coil. Functional data were acquired using a T2*-weighted gradient echo planar imaging sequence with the following parameters: 32 interleaved axial slices, repetition time = 2000ms, echo time = 30ms, flip angle = 90°, slice thickness = 3mm, field of view = 192x192, matrix 64x64). The first 10 time points were removed to allow magnetization equilibrium. T1-weighted scans were acquired to confirm no participants had brain abnormalities and for normalization with the following parameters: repetition time = 7.8ms, echo time = 3ms, flip angle = 20°, slice thickness = 1.13 x 1.13 x 1.0 mm, field of view = 290 x 290 x 176, matrix = 256 x 256 x 176. The scanning session involved a 7 minutes resting state scan (eyes open, fixating on a black cross on grey background) followed by two task runs each lasting approximately 15 minutes. Between the two sessions participants were given a short break. Finally, we recorded a seven minute structural scan.

### Pre-processing

#### Task-based fMRI

Pre-processing of the task based fMRI data was performed using Statistical Parametric Mapping (SPM8 [52]; available at: http://www.fil.ion.ucl.ac.uk/spm/software/spm8) implemented in Matlab R 2013a (The Mathworks Inc.; available at: http://www.mathworks.com). Data underwent the following processing steps: (1) slice-time correction, (2) motion correction, (3) co-registration of the T1-weighted image to the mean EPI scans, (4) normalized to MNI space using the T1-weighted normalization parameters computed during unified segmentation, (5) resampled to 2mm isotropic voxels, and (6) smoothed using a 6mm FWHM Gaussian kernel.

#### Resting state fMRI

Pre-processing of the resting state data used the DPARSF v2.3 toolbox [53] implemented in Matlab R 2013a. Data underwent the following processing steps: (1) slice-time correction, (2) motion correction, (3) co-registered the T1-weighted image to the mean functional image, (4) normalized to MNI space using the T1-weighted normalization parameters computed during New Segment and DARTEL, (5) resampled to 2mm isotropic voxels, and (6) smoothed using a 6mm FWHM Gaussian kernel, (7) nuisance regression using the six movement parameters, the signal from the the white matter and the signal from the CSF, and (8) band-pass filtered .008 to .01Hz.

### First level analysis

#### Task based fMRI

**Fig 2:**
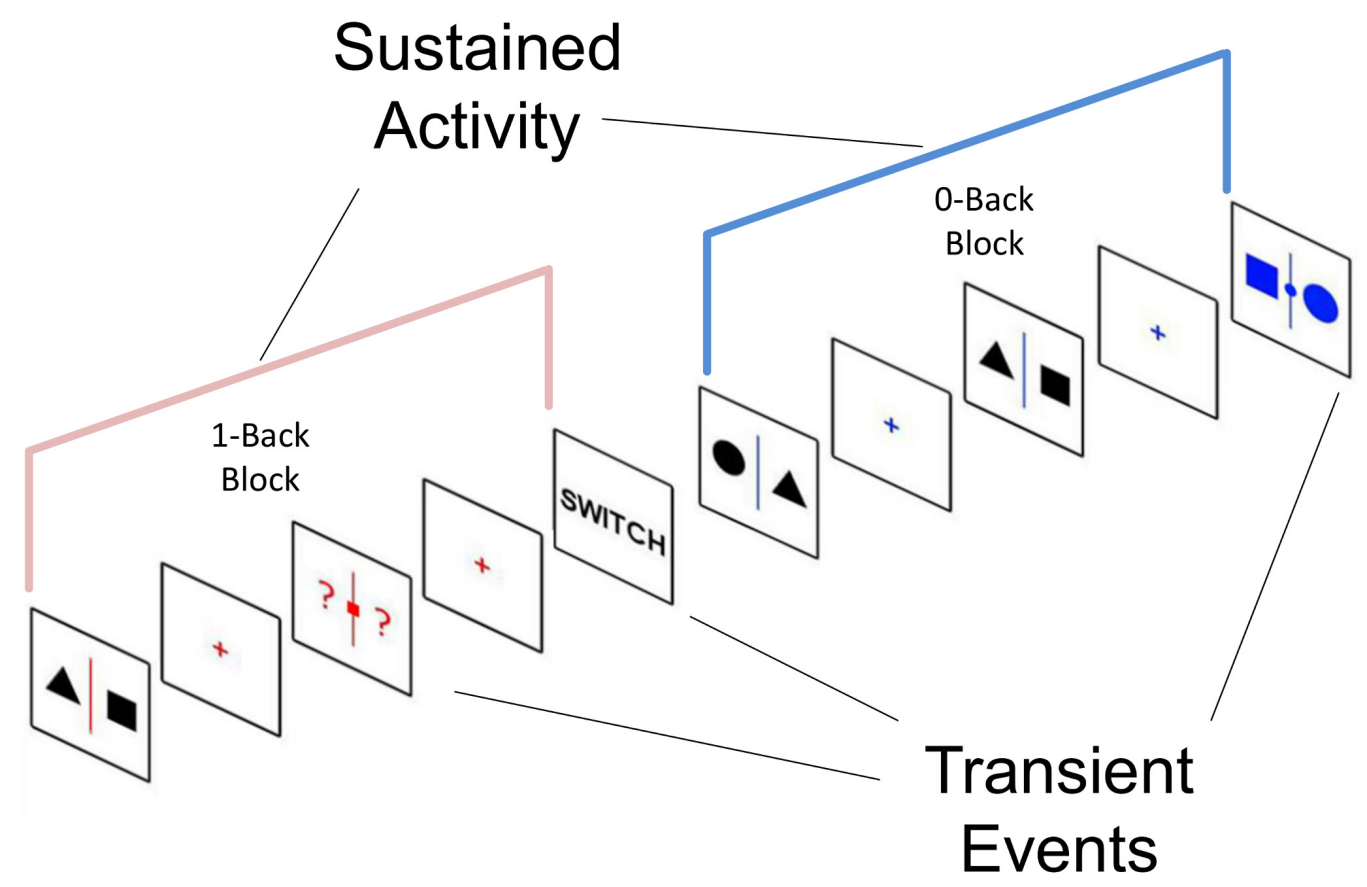
Analytic strategy. The task-based data was analysed using a mixed block / event design. We modelled transient events in both tasks as single event with a length equivalent to the stimulus duration. To model the sustained changes that occur during each task block we created a box-car that began at the beginning of the task block and lasted until the participant received the instruction to switch to the other task.

To analyse our mixed block-event related design (Fig 2) [54,55], we employed a GLM to model each event type. The transient events in each task (targets, switches and stays) were modelled as single events with the relevant duration in seconds (2, 4 and 4 respectively). The sustained activity in each task was modelled by creating a block that began at the first NT in each block and lasted until the participant switched to the other task. Each of these events was convolved with the canonical haemodynamic response function as implemented in SPM 8. The GLMs included a constant term per run, a high frequency signal filtering (cut off = 128 s), an AR(1) filter and the motion parameters. For each individual we computed two contrast images: (1) 1-back greater than 0-back sustained responses and (2) 1-back greater than 0-back target transient responses.

#### Resting state fMRI

To provide a quantitative description of the spatial extent of the DMN we calculated the functional connectivity of the PCC region using the resting state scans acquired as part of this study. We calculated the time series of two 6mm diameter spheres centred on the co-ordinates of the PCC [–8, –52, 26] in each hemisphere reported by Andrews-Hanna et al. [56] and used these as regressors in a standard functional connectivity analysis implemented using the DPARSF toolbox for SPM. Correlation coefficients were then transformed to Z-scores using Fisher’s r-to-Z transformation for group level analyses.

### Group Level Analysis

#### Task-based fMRI

One-sample t-tests were used to evaluate the differences in 1-back and 0-back BOLD responses for transient and sustained events separately. Results were masked with the grey matter mask provided by DPARSF. We used a voxel wise threshold of p < 0.01 and topological False Discovery Rate with a p-value of p < 0.05 as implemented in SPM to control for multiple comparisons.

#### Resting-state fMRI

A one-sample t-test was used to determine the extent of the DMN. The subsequent images were thresholded with a T of 7 and binarised. This was used as a mask for analysis of the task based data.

#### Conjunction analysis

One-sample t-tests were used for the transient and sustained events on the 1-back and 0-back conditions using a cluster-forming threshold of p < 0.05. We then calculated the overlap between the resulting maps and masked them with the DMN mask obtained from the resting-state analysis.

## Results

### Behavioural

**Fig 3:**
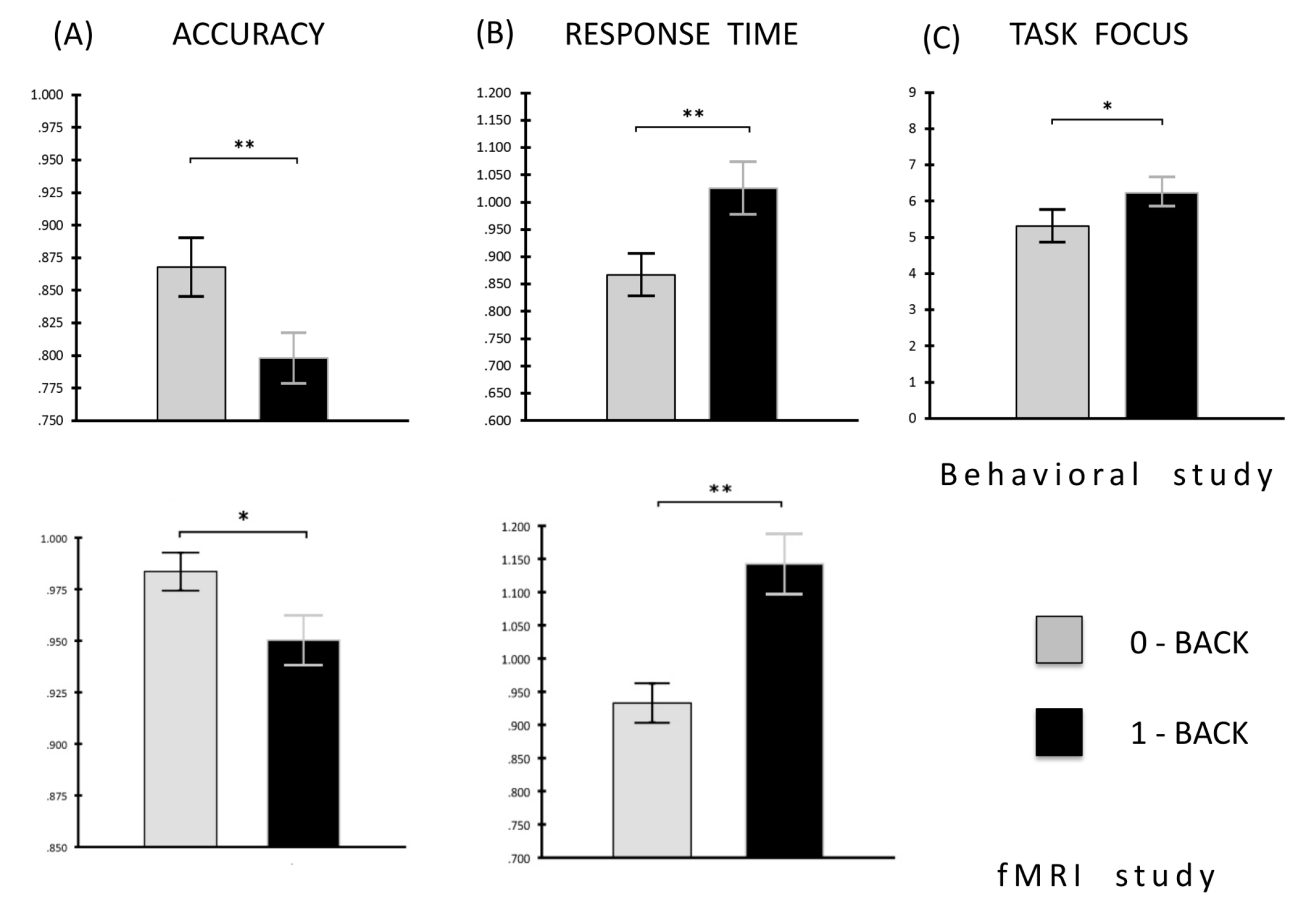
Behavioral results. Analysis of the behavioural data collected during both the behavioural (top three graphs) and fMRI (bottom two graphs) experiment indicated that participants were both faster (shown in ms) and more accurate when they were asked to make decisions about the location of a shape based on the present trial relative to where it was on the previous trial. In addition, analysis of the experience sampling data recorded in the behavioural experiment (top right graph), demonstrated that participants engaged in more off task thought during the 0-back than the 1-back task: participants rated their task focus on a scale from 0 (completely off task) to 9 (completely on task). The mean of participants’ responses to the probes in each condition is shown. ** p < 0.001, * p < 0.05.

We analysed accuracy and response time data for both the behavioural and neuroimaging samples using paired sample t-tests. These demonstrated that participants were both slower and less accurate in the 1-back than in the 0-back task in both experiments (behavioural RTs: *t* (25) = 3.75, p < 0.001; behavioural accuracy: *t* (25) = -4.92, p < 0.001; fMRI RTs: *t* (19) = 6.70, p < 0.001; fMRI accuracy: *t* (19) = 2.38, p = 0.03; see Fig 3A and 3B). In addition analysis of the experience sampling reports collected in the behavioural study indicated that participants rated their experience as reflecting greater off task thought in the 0-back than the 1-back task (*t* (25) = 2.45, p = 0.02; Fig 3 *C*).

### Functional Magnetic Resonance Imaging (fMRI)

#### Whole brain analysis–Transient activity

**Fig 4:**
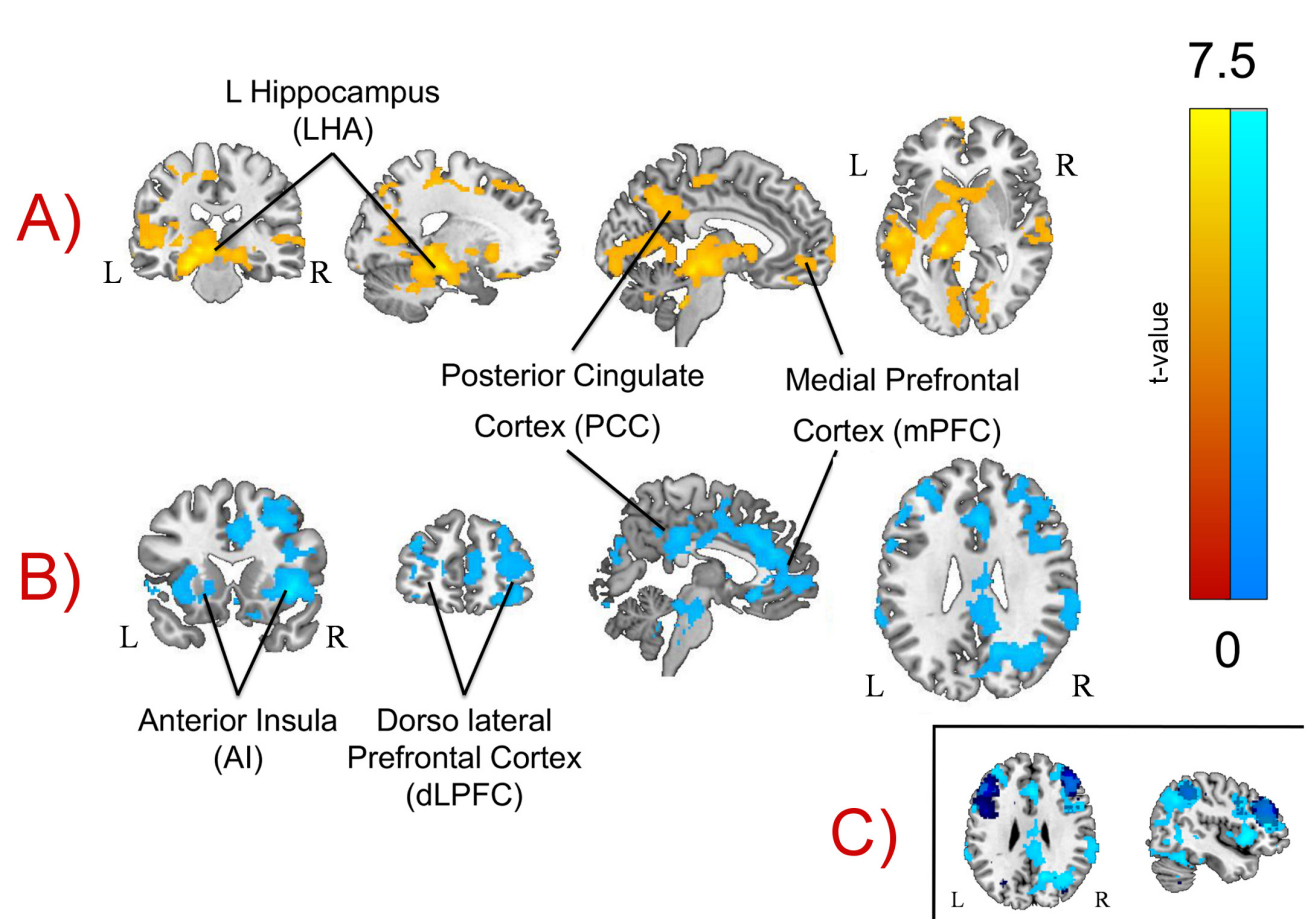
Whole brain analyses. We conducted a whole brain analysis of the observed transient and sustained changes in the BOLD signal. Row A): Yellow areas show sustained activation for the 0-back task. Row B): Blue areas show transient activation for the 1-back task. Importantly regions of both the medial prefrontal cortex and the posterior cingulate cortex exhibited greater activity during target retrieval in the 1-back task (B) and increased sustained activity in the 0-back condition (A). These images were created using a cluster forming threshold of p < .01 and multiple comparisons were controlled for using topological FDR (p < .05). Subpanel C) on the bottom-right shows the overlap in frontal and posterior dorsal regions between the transient activation for the 1-back task (light blue) and a meta-analysis using the term “working memory” using Neurosynth (dark blue).

We identified a large set of regions that were significantly more activated for correct responses to the targets in the 1-back task than the 0-back task (see Table 1 and Fig 4). These included regions traditionally associated with working memory including the anterior cingulate cortex, the anterior insula (bilaterally), inferior parietal sulcus (IPS) and regions of the lateral pre-frontal cortex (bilaterally). To ascertain whether this pattern of activity is consistently observed in working memory tasks we explored the overlap between our findings and those observed in a meta-analysis of studies involving the term working memory using Neurosynth [57]. We saw overlaps in regions of mid cingulate cortex as well as dorsal regions of lateral pre-frontal and parietal cortex. This information is presented as a sub panel in Fig 4.

**Table 1:** Transient activity. Regions showing increased transient BOLD activity during correct responding in the 1-Back > 0 Back task.

Region	Peak T	Peak equivZ	Peak p(unc)	x,y,z (mm)
Precuneus	5.27	3.85	0.001	14–64 30
	4.65	3.56	0.001	18–52 16
	4.62	3.54	0.001	10–46 0
Right Dorsolateral Prefrontal Cortex	5.05	3.75	0.001	32 14 42
	4.47	3.46	0.001	22 42 40
	4.22	3.33	0.001	28 20 46
Medial Prefrontal Cortex	4.98	3.72	0.001	0 36 22
	4.83	3.64	0.001	16 64 0
	4.72	3.59	0.001	28 56 8
Right Inferior Parietal Lobule	4.8	3.63	0.001	44–58 36
	4.6	3.53	0.001	36–62 26
	4.33	3.39	0.001	40–68 44
Posterior Cingulate Cortex	4.54	3.5	0.001	2–32 36
	4.01	3.22	0.001	10–46 34
	3.79	3.09	0.001	8–16 30

More relevant to the current investigation was the enhanced activity for targets in the 1-back task that extended into regions of the core DMN including the PCC and mPFC (Fig 4). We found no region exhibiting activity surpassing the cluster-forming threshold for the opposite contrast (0-back > 1-back).

#### Whole brain analysis–Sustained activity

The easier 0-back task activated areas of the DMN to a greater extent than in the harder 1-back task (see Table 2 and Fig 4). These included anterior and posterior regions of the mPFC and the PCC, as well as regions in the temporal parietal junction and the lateral temporal lobes. Activity was also enhanced in the several sub-cortical structures including the caudate / putamen, thalamus, hippocampus. We found no region exhibiting activity surpassing the cluster-forming threshold for the opposite contrast (1-back > 0-back).

**Table 2:** Sustained activity. Areas showing greater sustained BOLD activity in the O-Back than 1-Back blocks.

Region	PeakT	Peak Z	Peakp(unc)	x,y,z (mm)
Left Hippocampus	7.7	4.74	0.001	-24–20–18
	5.76	4.06	0.001	-22–34–8
	5.63	4	0.001	-16–34 2
Left Middle Temporal Gyrus	5.2	3.82	0.001	-54–8–18
	4.06	3.24	0.001	-62–18–10
	4.03	3.23	0.001	-52–16–22
Left Inferior Parietal Lobule	4.65	3.56	0.001	-38–72 36
	4.29	3.37	0.001	-48–62 42
	4.24	3.34	0.001	-38–74 28
Posterior Cingulate Cortex	4.59	3.53	0.001	-8–60 38
	4.02	3.23	0.001	-10–38 28
	4	3.21	0.001	-2–48 34
Retrosplenial Cortex	3.86	3.13	0.001	-6–66 6
	3.49	2.91	0.002	-18–66 18
	3.32	2.81	0.003	-18–64 8

#### DMN Region of interest analysis

**Fig 5:**
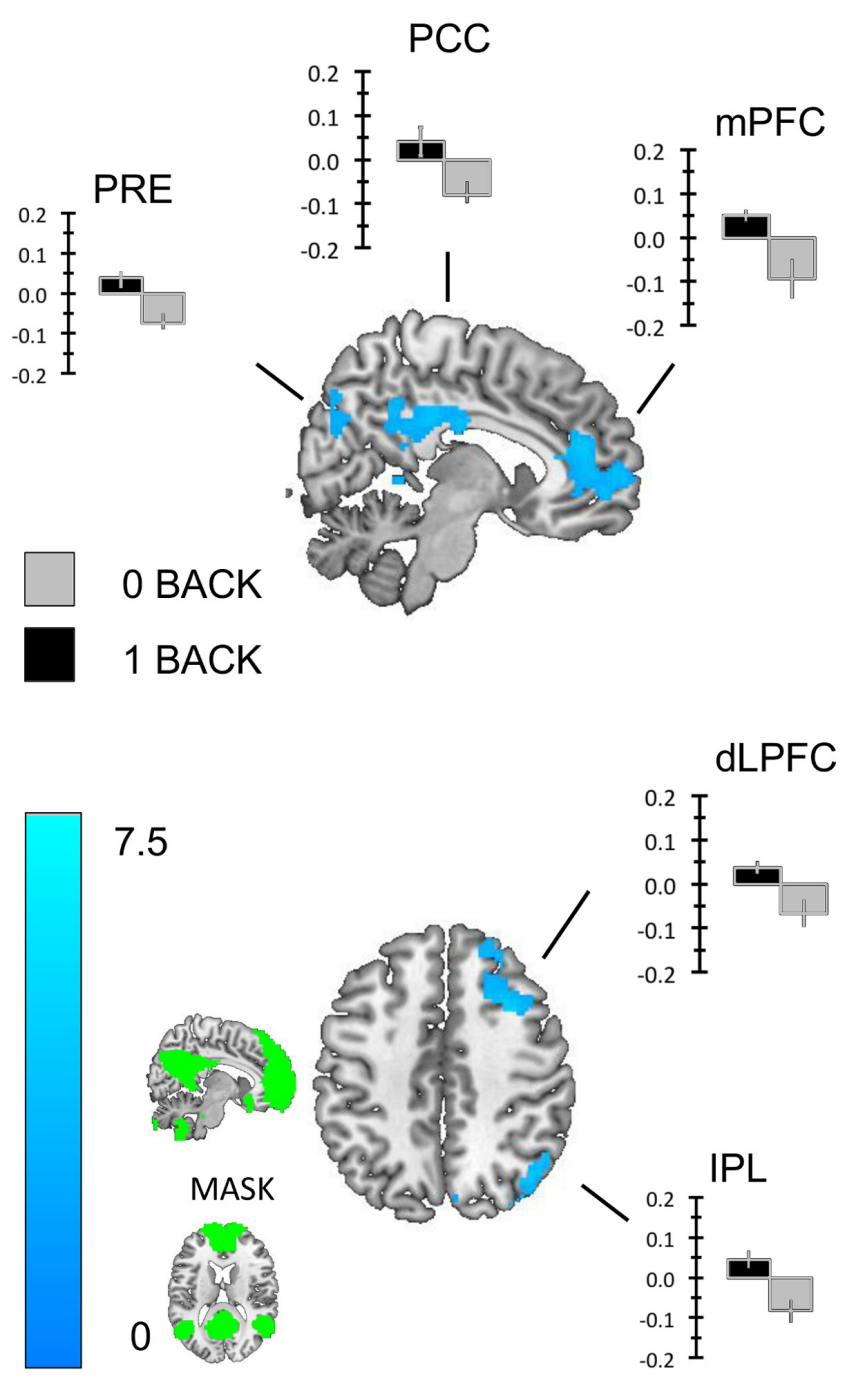
Transient changes with the Default mode network. We repeated the analysis using a mask of the DMN created using functional connectivity from a sample of 39 healthy participants. This analysis revealed clusters in the posterior cingulate cortex, regions of the ventral and dorso-medial pre-frontal cortex and the right tempo parietal junction. To identify whether these clusters of activity constituted increases in activity in the 1-back task we extracted the beta weights for each and plotted the group averages. These images were created using a cluster forming threshold of p < .01 and multiple comparisons were controlled for using topological FDR (p < .05). The image used as a mask is presented in the sub-panel.

**Fig 6:**
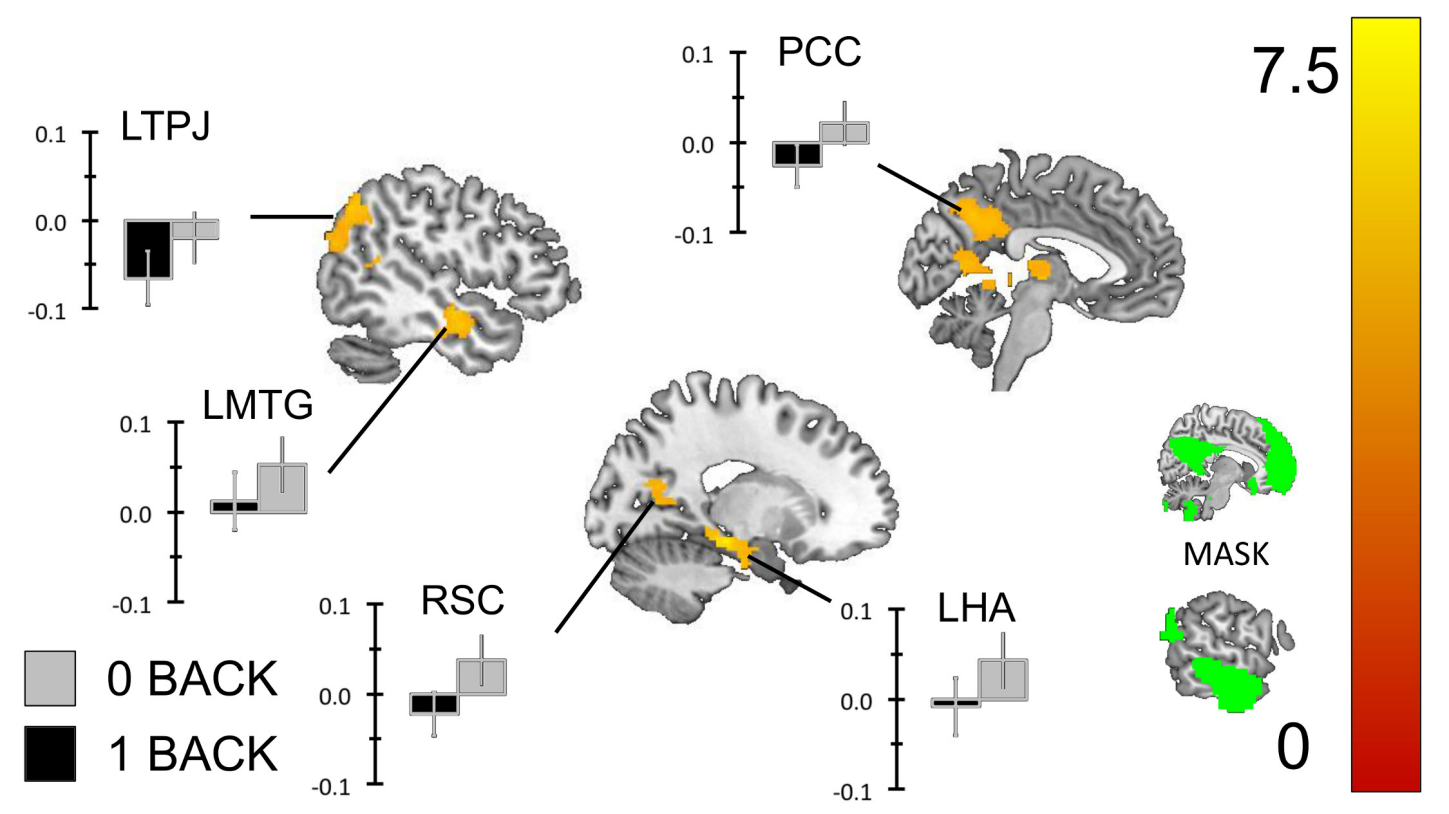
Sustained changes within the Default mode network. To identify which patterns of increased sustained activity in the 0-back task observed in the whole brain analysis we repeated the analysis using a mask of the DMN created using functional connectivity from a sample of 39 healthy participants. This analysis revealed clusters in the posterior cingulate cortex, the left hippocampus, the left middle temporal gyrus and the right tempo parietal junction. To identify whether these clusters of activity constituted increases in sustained activity in the 0-back task we extracted the beta weights for each and plotted the group averages. These images were created using a cluster forming threshold of p < .01 and multiple comparisons were controlled for using topological FDR (p < .05). The images used as masks are presented in the sub-panel.

We repeated the one-sample t-tests reported above using our mask of the DMN (see Fig 5). We found that regions of the precuneus (Prec), the PCC, the mPFC and regions of the right dorsomedial prefrontal cortex (dmPFC) exhibited greater transient activity for correct responses to the 1-back than the 0-back targets (see Fig 5). Similarly, regions of the PCC, the inferior parietal lobule (IPL), the left middle temporal gyrus (L. MTG) and the hippocampus (Hipp.) exhibited greater sustained activity in the 0-back than the 1-back task (see Fig 6). To demonstrate that these transient and sustained changes constitute increases in the BOLD signal we extracted beta weights from each cluster using the rfxplot toolbox for SPM (Figs 5 and 6).

#### Conjunction analysis

**Fig 7:**
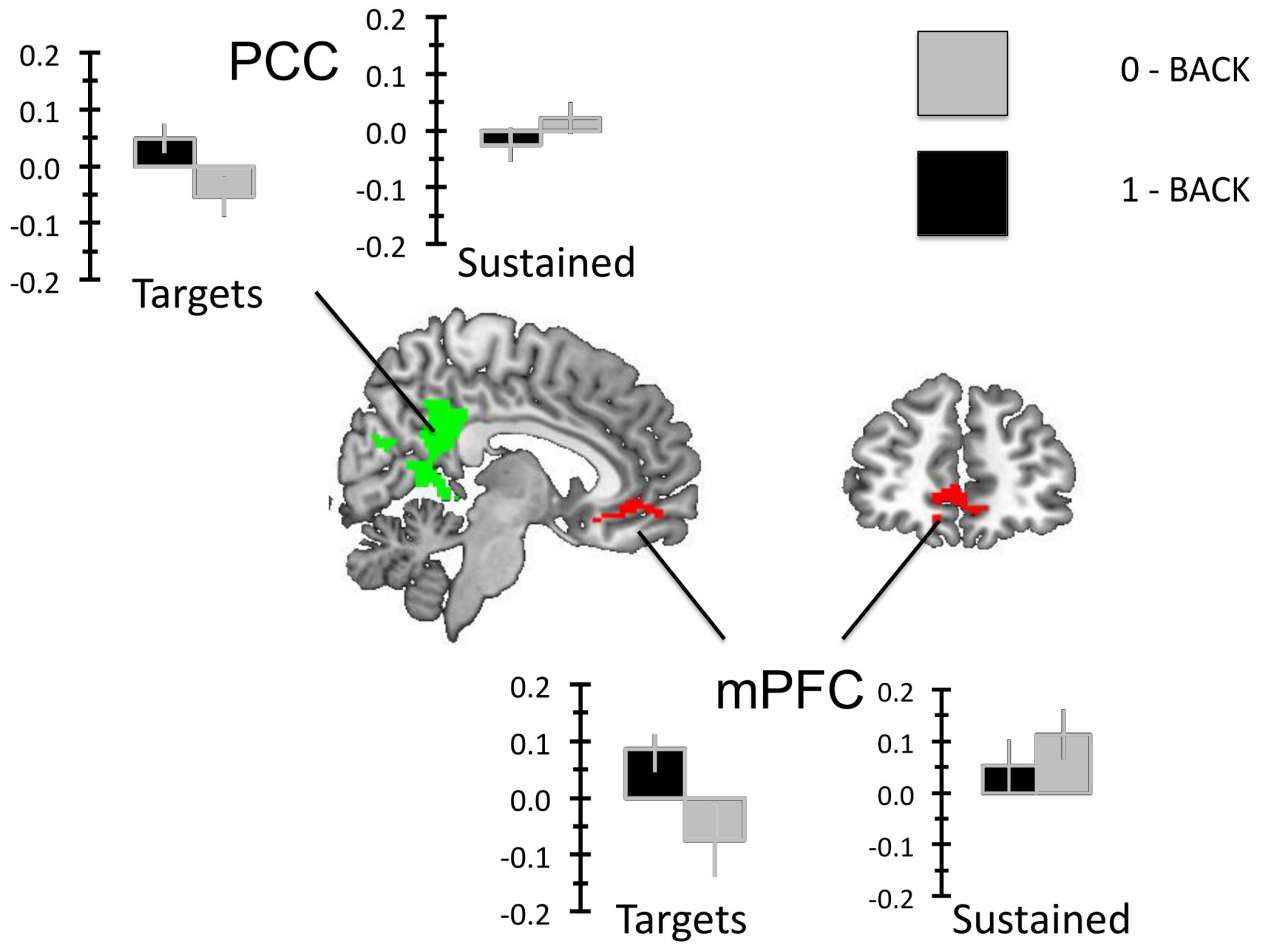
Spatial conjunction analysis. To formally compare the activations arising from the sustained and transient increases in DMN activity observed in this experiment we created whole brain images for the Targets (1-back > 0-back) and the Blocks (0-back > 1-back). This analysis used a cluster forming threshold of p < .05 controlling for multiple comparisons using FDR (p < .05). These images were binarised and we calculated their overlap with each other as well as the DMN mask used in the prior analyses using ImCalc function of SPM.

Finally we explored the spatial similarities in the sustained and transient changes in the DMN by examining their spatial conjunction. As the logic of conjunction of temporally different events is a relatively stringent statistical test we used a liberal cluster-forming threshold of p < 0.05 to rule out a Type II error. We calculated the overlap between the whole brain analysis of transient increases in the 1-Back task and the sustained increases in the 0-back condition using this liberal threshold. This image was masked by the DMN mask generated from the resting-state fMRI study. This analysis revealed patterns of cluster corrected sustained activity in the 0-back task *and* of target related activity in the 1-back task, which overlapped in a region of the PCC / retrosplenial cortex and in the mPFC (see Fig 7).

## Discussion

Using fMRI during performance of our working memory task, we found brain activity in working memory regions when participants performed a 1-back rather than a 0-back task, confirming previous findings. Importantly, however, these regions were accompanied by activity in regions within the mPFC and PCC corresponding to core hubs of the DMN. These results are inconsistent with at least two prevalent views of the functions of this network. Regions of mPFC and PCC increased activity when making a decision about a shapes position from memory and decreased activity when making the same decision using immediate perceptual input. As deciding where a shape *was* is more difficult than deciding where a shape *is*, the patterns of activation and deactivation of the mPFC and PCC in the more demanding 1-bask task indicates that this activity is *not* a task deactivation [38]. Furthermore, the relative activation of the mPFC and PCC by decisions regarding the position of a shape demonstrates that strong emotive or autobiographical ties with a stimulus are not necessary to activate these regions [24]. Nor must the stimulus be encoded in long-term memory [44] as is the case for a famous face: our study shows that this capacity to guide cognition based on information from memory is *not* equivalent to long–term memory because the core of the DMN was activated when decisions were made based on representations of information that was presented a matter of seconds ago. Instead these data can simply be accounted for by *the mnemonic facilitation hypothesis*: *that core regions of the DMN allow cognitive operations to be guided by information unrelated to immediate perceptual input*.

Further support for our hypothesis comes from the observation that overlapping regions of the PCC and mPFC exhibited *sustained* activity in the 0-back task as well as *transient* target related activity in 1-back task. Our experience exampling study confirmed that the 0-back task was characterized by greater off task thought (see Fig 3) making it possible that the pattern of activity seen in both the PCC and mPFC may indicate a common role for these regions in allowing cognition the freedom to perform operations that are not constrained by immediate input whether it is to do with the momentary demands of a task or not. Without direct evidence linking DMN activity in the task to the experiences reported by the participants, this interpretation should be treated with caution; however, we hope to test this hypothesis in a larger sample of participants with online measures of self-generated thought in the future.

Our hypothesis that the DMN allows thought and behaviour to be guided by memory explains why this network is prominent in a range of higher order cognitive states such as future thinking, mentalizing or creativity, as well as task irrelevant activities such as daydreaming or mind wandering. All of these states depend on being able to consider information from memory, often to the extent that this can be detrimental to perceptual processing (as in the case of mind-wandering, [58–60]). Our hypothesis that the DMN allows thought to be shaped by representations from memory also explains why this network has an analogue in a wide range of non-human species. While it is a matter of debate whether complex abilities like mental time travel or language are unique to humans [61], the capacity to guide behaviour using information from memory is a universal feature of mammalian cognition. As our data suggests that the DMN can support relatively mundane cognitive processes in humans (“Which side was the triangle on?”) it seems that the presence of analogues for this network in different species may simply reflect the fact that they are also capable of guiding behaviour based on information other than immediate perceptual input.

Our hypothesis gains further support from work showing that perceptual input and DMN activity are often in opposition [16,62,63]. For example, the recent work of Huijbers and colleagues [16] showing DMN increases in episodic memory retrieval and decreases during episodic memory encoding. This suppression of the DMN during encoding is consistent with the reduction in sustained activity we observe in the 1-Back task because under these conditions participants must continually encode information from the environment. Our hypothesis also predicts that changes in the value of immediate input for a specific stimulus or task will be associated with increased activity in the DMN. This prediction is supported by a recent study of repetition suppression, which observed decreases in DMN deactivations during encoding as participants viewed the same items, suggest that the DMN deactivates less as participants form a stronger memory trace of a stimulus [62].

When participants made decisions in the 1-back task we also observed increased activity in cortical regions outside of the DMN. For example, we found increased activity in the DLPFC and IPS: both elements of the fronto-parietal network (FPN [64,65]). BOLD increases during 1-back decisions were also observed in the anterior insula and anterior cingulate, regions which are important in the cingular-opercular, or saliency network [66]. Variations on this pattern of network activity has been observed when participants make plans for their future [67–69], engage in creative thought [23], resist future rewards in the service of greater long term return [70], and when maintaining social information in memory [71]. Most recently, Spreng and colleagues demonstrated that the DMN and the FPN co-operate to perform a working memory task with famous faces as the target [44]. Our study, therefore, adds to a growing body of research that demonstrates that many complex higher order tasks cannot be attributed to a single neural network and instead depend on the coordinated activity of multiple networks in a flexible fashion (for further discussion see [42,72–74]).

Our data demonstrate that core regions of the DMN are activated when participants are asked *where a shape was* rather than *where it is right now*. A simple account of these data is that it reflects the role of the DMN in allowing cognition to be shaped by representations that are distinct from those provided by immediate perceptual input. We propose this process is *necessary* for a range of different functions including task judgements that depend on recollections based on memory but also daydreams about a holiday or ruminations about a personal problem, thus accounting in a parsimonious manner for many of the functions that utilize the DMN. While this hypothesis is important because it offers an account for why the DMN is common to seemingly disparate functions, it offers no explanation for how these functions are differentiated within the DMN, nor the precise mechanisms that allow behaviour to be guided by information that is represented internally rather in the external environment. Moving forward it is likely we will need more sophisticated models of the cognitive functions that the DMN supports, as well as more comprehensive accounts of the functions that different regions perform, in order to truly understand the complex role this network plays in human cognition.
